# Interactions of Genes and Sodium Intake on the Development of Hypertension: A Cohort-Based Case-Control Study

**DOI:** 10.3390/ijerph15061110

**Published:** 2018-05-30

**Authors:** Mei-Ling Chen, Tzu-Pi Huang, Tai-Wei Chen, Hsin-Hua Chan, Bing-Fang Hwang

**Affiliations:** 1 College of Human Science and Social Innovation, HungKuang University, No. 1018, Sec. 6, Taiwan Boulevard, Shalu District, Taichung 43302, Taiwan; mlchen@hk.edu.tw; 2 Department of Plant Pathology, National Chung Hsing University, Taichung 402 Taiwan; tphuang@nchu.edu.tw; 3 Department of Occupational Safety and Heath, College of Public Health, China Medical University, No 91 Hsueh-Shih Rd, Taichung 40402, Taiwan; u102014412@cmu.edu.tw (T.-W.C.); u9614406@cmu.edu.tw (H.-H.C.)

**Keywords:** G-protein beta3 subunit, sodium, hypertension, gene-environment interaction, modifier gene, cohort-based case-control studies

## Abstract

There have been few studies investigating interactions of G-protein beta3 subunit (*GNB3*) C825T (rs5443) and dietary sodium intake on the risk of hypertension, i.e., BP salt sensitivity. The study aims to evaluate joint effects of *GNB3* polymorphisms and sodium consumption on the development of hypertension. A cohort-based case-control study was conducted in 2014. There are 233 participants with newly diagnosed hypertension in the case group and 699 participants in the gender-matched control group. The primary outcome is the development of hypertension over a 10-year period. The determinants of hypertension were three genotypes of SNP in *GNB3* (TT; CT; and CC) and two dietary salt categories on the basis of the level of sodium consumption representing high (>4800 mg/day) and low-sodium (<2400 mg/day) diets. The development of hypertension increased with participants carrying TT genotype and high-sodium diets comparing with those carrying TC or CC genotype with low-sodium diets (adjusted OR 3.23, 95% CI 1.52–6.83) (Rothman synergy index = 3.79). The study suggests that *GNB3* C825T polymorphism may influence the response of the renin-angiotensin system to high-sodium diet. It implies that *GNB3* can be served as an easy, inexpensive, and early genetic marker of salt sensitivity to blood pressure. Salt-sensitive individuals should pay more attention to salt intake to reduce cardiovascular morbidity or mortality.

## 1. Introduction

Hypertension remains a major risk factor of cardiovascular disease morbidity and mortality. It has been recognized that both genetic and environmental factors play key roles in the causes of hypertension, as well as their interactions [1]. In a preventive perspective, it is important to have a better understanding of environmental, dietary, and behavioral factors. Moreover, identifying determinants of genetic susceptibility to those factors would be beneficial.

Several genes known or suspected to be involved in blood pressure (BP) pathways—such as renin-angiotensin system, neuroendocrine system, vasoactive peptides, nitric oxide, calcium and potassium channels, wnt/beta-catenin signaling pathways, kidney solute transport and kidney structure or proteins—have been identified in genome-wide association studies in the general population [2]. Intracellular signal transduction was considered as one of the most important pathways, contributing to the development of hypertension [3]. G proteins act as signal transducers which transmit signals from a couple of chemokines, hormones, neurotransmitters, and autocrine and paracrine factors [4]. A single nucleotide polymorphism C825T (rs5443), in exon 10, leads to an exchange at position 825 in the cDNA of the gene encoding the G-protein β3 subunit (*GNB3*) the amino acid cytosine by thymidine, which results in increased intracellular signal transduction between receptors and effectors. It seems reasonable to expect that the 825T allele is associated with increased risks of hypertension [5]. Siffert et al. (1998) reported the *GNB3* C825T polymorphism had an effect on essential hypertension [6]. Since then, there were positive or negative findings of different ethnic population in other epidemiological studies. A critical review of 34 studies composed of 14,094 hypertensive cases and 17,760 controls suggested that C825T gene polymorphism increased risks of hypertension, yielding an odds ratio (OR) of 1.08 (95%b confidence interval (CI): 1.01–1.15) for TT compared with the CC + CT genotypes, particular in Caucasian [7], but not in Chinese populations [8].

High sodium intake in the diet is one of the most important factor increasing risks of hypertension [9,10]. Understanding interactions of genes and dietary sodium on the BP may provide an important clue to develop new antihypertensive drugs targeting the molecular mechanisms which are related to sodium homeostasis and BP regulations for clinical management of patients [11]. Individuals who are sensitive to salt are more likely to develop hypertension than those who are resistant to salt. However, early genetic markers of the renin-angiotensin system to high-sodium diet implications for diagnostic presumptions of BP salt sensitivity have not been explored yet [12].

There were limited epidemiological studies investigating gene–sodium interactions between *GNB3* C825T and dietary sodium intake on the development of hypertension and somehow provide controversial results [13,14]. Further studies are needed to clarify this topic. To evaluate the relation between dietary sodium intake and the development of hypertension, we conducted a cohort-based case-control study in 2014. This design enabled us to confirm a suitable temporality for the hypothesized exposure and outcomes. Furthermore, the study tested the hypothesis that possible joint effects of *GNB3* and dietary sodium intake on the risk of hypertension is remarkable comparing their independent effects. This study assumed that participants with *GNB3* TT genotype increased their susceptibility to the effects of dietary sodium consumption on the development of hypertension. Identifying individuals who are BP salt sensitivity helps us to provide an efficient means of prevention so that they could benefit most from low-sodium dietary intervention.

## 2. Material and Methods

### 2.1. Study Population

Shalu is a town in Taichung city, Taiwan. It is assumed that the characteristics of its population (approximately 30,000) would be appropriate for a hypertension study and would facilitate follow-up of participants. Residents were considered eligible between the age of 25 and 45. We distributed informed consent forms and baseline questionnaires in June 2004 as the cohort entry. The study population at the baseline consisted of 1301 men and women without hypertension at entry. In June 2014, a 10-year follow-up survey was conducted to all participants of the cohort. We received completed questionnaires, physical examinations, assessments of dietary sodium intake and blood samples from 1179 participants (1179/1301 = 90.62% of the baseline study population). We excluded participants suffering from hypertension or taking antihypertensive medicines at the baseline health check-ups (*n* = 52). Therefore, the total study population is 1127 participants at the cohort entry (baseline). In October 2014, a cohort-based case-control study was conducted. We identified 233 new cases developing hypertension during the study period from 15 June 2004 to 31 June 2014. One to three frequency matching on gender was performed and 699 normotensive controls from 2004 baseline survey was selected. The final study population consisted of 233 cases and 699 controls.

### 2.2. Data Collection

Participants were asked about personal background, health status, lifestyle, other relevant risk factors and health check-ups in the baseline survey. Some questions on cardiovascular health status were from the Taiwan Heart Association and American Heart Association. Two cardiac physicians helped to modify questions to correspond to the daily language usage. The identical questions about health and environment were used in the baseline and follow-up survey. The study protocol was approved by the Institutional Review Board of China Medical University Hospital and it conformed to the principles outlined in the Helsinki Declaration. Written informed consent was obtained from each subject (CMUH105-REC3-033). 

### 2.3. Definition of Hypertension

The study aimed to investigate the development of hypertension during the study period. We defined hypertension as people with newly doctor-diagnosed high blood pressure or receiving any kinds of antihypertensive medicine during the study period or having a resting systolic blood pressure of ≥140 mm Hg or a resting diastolic blood pressure of ≥90 mm Hg in the follow-up physical examination in 2014. We only included participants without hypertension or not taking antihypertensive medicine at baseline in the analyses. Afterwards, we identified those who indicated the development of hypertension during the 10-year study period.

### 2.4. GNB3 C825T Genotyping

The DNA Blood Mini Kit by QIAGEN^®^ (Hilden, Germany) was used in genomic DNA extraction. The detailed description of the genotyping could be referred to in Shiffert’s paper [6]. In brief, the restriction enzyme *Bse*DI (fermentas) was used to digest the PCR products. The *GNB3* C825T polymorphism was visualized as 116 bp and 152 bp fragments for CC genotype; 268 pb fragment for TT genotype; and 116 bp, 152 bp, 268 pb fragments for CT genotype. A laboratory assistant blind to the clinical status of individual participants performed all assays and obtained genotype assignments following two accordant experimental results. We sequenced approximately 15% of random samples directly. Moreover, all of them were consistent with previous genotyping results.

### 2.5. Assessment of Sodium Intake

The sodium intake was assessed by using a questionnaire according to responses to the following questions: “How often is sodium added in your cooking or preparing foods? The answer is never, rarely, occasionally or very often?” If the participant answered “rarely” or “never”, the amount of optional sodium used in the recipes was deleted; if the participant answered “occasionally”, one-half of the optional sodium used in recipes was also deleted [15]. Information on sodium used in food preparation and sodium added at the table was from the amount of self-reported teaspoon salt per day at the baseline survey. The Department of Health in Taiwan issued recommended dietary guidelines. The current guidelines specify a maximum daily sodium intake of 2400 mg for young healthy people. High sodium-intake participants were defined as those who reported to intake sodium greater than two teaspoons of sodium per day (>4800 mg/day), whereas a low sodium intake would be at or below 2400 mg/day—roughly the amount a teaspoon of sodium. Therefore, we constructed two categories of sodium diets on the level of sodium consumption representing high (>4800 mg/day) and low sodium intake (≤2400 mg/day).

### 2.6. Covariates

The covariates used in the analyses included: gender, age, body mass index (BMI; weight-kg/height-m^2^), smoking, drinking, low-density lipoprotein (LDL; mg/dl), high- density lipoprotein (HDL; mug/dl) and triglyceride level (TG; mg/dl), creatinine (mg/dl), uric acid (mg/dl), diabetes mellitus and thyroid disease. Age at the baseline survey was categorized in four dummy variables (41–45, 46–50, 51–55, >55 years, and <40 years as reference category) to allow adjustment of non-linear trend. Smokers were defined as those smoking cigarettes more than three days per week in the past six months; alcoholic consumer were defined as those consuming alcohol during the past 30 days. Exercisers were defined as those who exercise per week. Exercise frequency per week was categorized as <1 h; 1–2 h; 3–4 h and > 5 h (reference). Fruit and vegetable intake was grouped in three categories: high (71–100%), median (51–70%), and low (<50%) dietary vegetable consumption. The level of creatinine and uric acid was divided into two categories (abnormal: <0.6 or >1.4 mg/dl, normal: 0.6–1.4 mg/dl; abnormal: <2.5 or >7.5 mg/dl, normal: 2.5–7.5). The urine protein was labeled as positive, suspected (+/−) or negative.

### 2.7. Statistical Methods

First, the odds ratio of hypertension was estimated based on three genotypes of SNP in *GNB3* (TT, CT, and CC) and two dietary sodium intake categories. We adjusted odds ratios using the aforementioned covariates by applying multiple logistic regression analysis.

Second, synergistic effects of *GNB3* and two indicators of dietary sodium intake on the risk of hypertension were studied. The odds ratios (OR) of hypertension in the following four categories were compared: (1) CT or CC genotype and low sodium intake (OR00, reference category); (2) TT genotype and low sodium intake (OR10); (3) CT or CC genotype and high sodium intake (OR01); and (4) TT genotype and high sodium intake (OR11). Then we derived their odds (O) in four categories (O00, O01, O10, and O11) from the same logistic regression model and adjusted for the covariates in the following formulate.

Logit (p) = α + β_1_*GNB3* + β_2_Dietary Sodium Intake + β_3_Age (41–45 years) + β_4_Age (46–50 years) + β_5_Age (≥51 years) + β_6_BMI + β_7_LDL + β_8_HDL + β_9_TG + β_10_diabetes mellitus + β_11_Smoking + β_12_Alcoholic Consumer

Synergistic effect was calculated using the attributable proportion (AP) by substituting odds ratios for risk ratios adjusting for confounding factors, which was suggested by Andersson et al. 2005 [16].

$$\mathrm{AP}=\frac{{\mathrm{OR}}_{11}-{\mathrm{OR}}_{10}-{\mathrm{OR}}_{01}+1}{{\mathrm{OR}}_{11}}$$

Finally, we used the Rothman synergy index calculated by substituting odds ratios for risk ratios and its 95% CI to assess the synergistic effect of the two factors [17]. The following formula was used to calculate the synergy index:

$$S=\frac{{\mathrm{OR}}_{11}-1}{\left({\mathrm{OR}}_{10}-1\right)+\left({\mathrm{OR}}_{01}-1\right)}$$

A synergy index greater than suggested a synergistic effect. The 95% CIs of S values were calculated by their S values and variance covariance matrix [18]. We further performed sensitivity analysis focusing on nonsmoking and nondrinking participants to reduce discrepancy.

## 3. Results

### 3.1. Study Population

A total of 233 participants (25.5%) suffered from hypertension when joining the study. Table 1 compared the demographic features of participants in the control and case groups at baseline. The participants in the case group were older, heavier, higher TG, lower HDL; and there were more smokers, alcohol consumers, and diabetics comparing with those in the control group.

### 3.2. Independent Effects of GNB3 and Sodium Intake

*GNB3* TT genotype was related to the development of hypertension with an adjusted odds ratio of 1.21 (95% CI 0.85–1.74), comparing with CT + CC genotype (Table 2). Table 3 showed the crude odds ratio for hypertension based on two indicators of dietary sodium intake and adjusted odds ratio comparing with the reference category. The risk of hypertension was associated with the high sodium intake (adjusted odds ratio: OR 1.88 and 95% CI 1.20–2.94).

### 3.3. Joint Effects of GNB3 and Sodium Intake on the Risk of Hypertension

**Figure 1:**
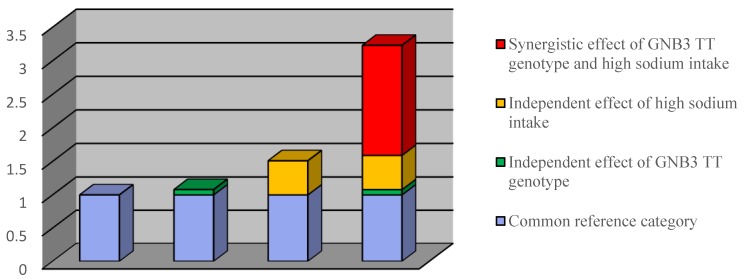
Odds ratio with contributions from different *GNB3* gene polymorphisms or sodium intake categories.

In Table 4, it presented the odds ratios of hypertension in four categories indicating the reference, independent effects of *GNB3* and dietary sodium intake and their synergistic effects. Participants with high sodium intake and *GNB3* (CT and CC combined) increased the risk of hypertension significantly with an adjusted odds ratio of 1.51 (95% CI 0.88–2.60) (Table 4). Effects of healthy sodium intake in participants with TT genotype also increased with an OR of 1.08 (0.72–1.61). Comparing with participants in the reference category, the adjusted OR of hypertension for participants with both TT genotype and high sodium intake was 3.23 (95% CI 1.52–6.83). Therefore, the attributable proportion due to interactions (AP) between high sodium intake and TT genotype and was 51.0%. Furthermore, we calculated the Rothman synergy index (s) 3.79 (95% CI 0.58–24.66) greater than 1, but the confidence interval shows a little wider. The result indicated additive interactions between *GNB3* C825T and dietary sodium intake. Figure 1 demonstrated additive interactions between *GNB3* gene polymorphisms and sodium intake in terms of synergism effects marked by red bar. We further focused on nonsmoking and nondrinking participants in the Supplement Table S2. It showed stronger synergistic effects of *GNB3* C825T and dietary sodium intake on the development of hypertension (s = 5.22) (Supplement Figure S1).

## 4. Discussion

Participants with *GNB3* TT genotype had more than 21% increased risk of developing hypertension, but this was not statistically significant. The high sodium intake increased the risk of hypertension by 88%. Furthermore, the results indicated that synergistic effects of genetic susceptibility *GNB3* and high sodium intake were greater than expected independent effects on an additive scale.

### 4.1. Results Validity

A cohort-based case-control study provided a suitable method to evaluate the role of dietary sodium intake in the development of hypertension. We have followed 90.4% of the 1301 participants for 10 years. The selection bias may not be an issue because the distributions of the population characteristics at cohort entry were similar to those in the 10-year cohort and there were only 122 (9.6%) participants lost to follow-up (supplement Table S1). A prospective study minimized information bias. We further expanded the ratio of case and control as 1:3 to reach optimal sample size. The final study population consisted of 932 subjects. Although the power calculation for logistic regression with binary interaction was up to 0.786 [19], the small sample size error should be minimized. Since the study was based on limited sample size for detecting gene–sodium interactions and adjusting for eight confounding factors, the results should be interpreted cautiously. We further validated the estimation of sodium intake in the questionnaires by assessing urinary sodium excretion collecting two 24-h urinary specimens at each of 50 random sampling subjects, approximately 5% of the study population (*n* = 932), along with 24-h dietary sodium recall. The correlation between daily dietary sodium intake and mean of two 24-h urinary collections for sodium was 0.75 (95% CI = 0.53–0.84). The reproducibility, namely the coefficient of intra-variation and inter-variation were less than 2.2% and 2.6%, respectively. Therefore, the estimation of sodium intake in the questionnaires was valid and reliable. Due to the lack of assessing dietary sodium intake over time, it may introduce imprecision of salt measurements. The gap of the lack of personal salt measurements was filled with some strengths in assessing sodium intake. We assessed dietary sodium intake before the onset of hypertension and most of the participants rarely changed their habit of sodium intake. However, we still cannot exclude the possibility that higher self-reported sodium intake was associated with the development of hypertension.

The outcome assessment in this study was based on JNC 7 criteria without ambulatory blood pressure measurements as most previous studies. Misclassification might be random and thus resulted in the underestimation of true effects. Misclassification could come from different diagnostic criteria, compromised identification of new hypertension cases, and wrong questionnaire information answered by participants. The Taiwan National Health Insurance, which is an affordable public health care system, provided patients fulfilling their diagnostic criteria for hypertension with 90% reimbursement for hypertension medication. The National Health Insurance Bureau was responsible for approving diagnoses when patients applied for subsidies and that will reduce the problem of diagnostic heterogeneity. All hypertension cases indicated in the questionnaires were verified by physicians.

To avoid over-adjustment for multiple covariates, we further performed stepwise regression analyses (*p* < 0.15) to get the parsimonious model. In the multiple logistic regression analysis, we took most known potential confounders—such as demographic characteristics, regular exercise, alcohol consumption, smoking, diabetes mellitus, thyroid disease, fruits and vegetable intakes, creatinine, urine acid, urine protein, LDL, HDL, and TG—into consideration. However, other factors, such as sleeping habits, types of work (night shift), variability of weight throughout the observation period, insulin assays for insulin resistance, physical activities, statin used, excessive drinking beyond gene polymorphism and sodium intake could have contributed to the development of hypertension. We cannot rule out the possibility that these residual confounding may bias the association.

Unbiased genetic approaches, especially genome-wide association studies (GWAS) have identified novel genetic targets in the pathogenesis of hypertension, but so far these target account for only a small proportion of heritability of hypertension. Characterization of hypertension including predictive genetic markers will lead to an improved understanding of disease heterogeneity contributing to the development of new target treatment. Considering there was no significant relationship between *GNB3* C825T and hypertension, one limitation was that we only tested one SNP within the gene. Although our result was consistent with previous two Japanese studies [20,21] and GWAS studies [22], it was difficult to take the true-positive of our findings unless we validated the findings in an independent study for replication. We cannot rule out this limitation in our study.

There existed an existing debate if the fitting statistical model should be used to determine the scale or an additive scale can be used to assess the interaction irrespectively of the underling statistical model. It was recommended that the additive scale be more suitable to assess synergistic or antagonistic effects [23]. The important manner for reporting the additive interaction is it goes well with the sufficient-component cause model [24]. Also, it may be more appropriate to use the additive scale using absolute risks in clinical or public health decision making [25].

### 4.2. Synthesis with the Previous Knowledge

Two studies found no apparent evidence that *GNB3* C825T variant played an important role in BP salt sensitivity [13,14]. In a study conducted by Pamies-Andreu et al., the subjects were 102 patients with hypertension in Spain and the findings indicated no association between salt sensitive hypertension and *GNB3* C825T [13]. Gonzalez-Munez et al. conducted a study of 46 patients with hypertension in Barcelona to elaborate association between *GNB3* C825T and BP salt sensitivity. They indicated that *GNB3* C825T polymorphism did not have a major effect on the pressor response to salt in hypertension [14].

Identification of individuals who are salt-sensitive or salt-resistant are crucial for preventive and personalized medicine in the treatment of hypertension. Our results provide a strong gene–sodium interaction between *GNB3* C825T and dietary sodium intake on the development of hypertension. The results also support the usefulness of early genetic marker of BP salt sensitivity. It is possible that our results in Taiwanese cohort are different from studies in Spain due to race specific. The pathophysiologic mechanisms of *GNB3* C825T on BP salt sensitivity are complicated and known in part only. It is possible owing to their biological influences on sodium homeostasis via renal tubular sodium reabsorption and sodium-proton exchanger, respectively [12]. Further studies are warranted to replicate this novel finding in other populations. However, our study assessing the gene–sodium interactions may limit extrapolation of results to populations with other genetic, behavioral, and environmental factors.

## 5. Conclusions

Our results strengthened the evidence that high-sodium intake increased the risk of the development of hypertension. In the previous studies, the relation between *GNB3*, sodium intake, and the risk for developing hypertension were obtained mainly by cross-sectional designs for establishing causal relationships which cause the most important threat against validity. In our cohort-based case-control study, the threat to validity can be avoided. In addition, our result suggested that gene–sodium interactions play an important role in the risk for developing hypertension i.e., participants with both *GNB3* TT genotype and dietary high sodium are more susceptible to developing hypertension. Identifying individuals who are at high risk of hypertension is helpful in providing them an efficient means of prevention. Individuals who are salt-sensitive should pay more attention to dietary sodium intake to reduce their risk for hypertension. They should monitor their sodium intake and limit high-sodium consumptions in their diet. Furthermore, they would get advantages most from more intensive reduction in sodium intake [26]. Since the gene-environment and gene-gene interactions on the risk for hypertension may also be intricate and have important clinical implications, further longitudinal studies are warranted to replicate this novel finding.

## Figures and Tables

**Table 1:** Baseline population characteristics.

Characteristics	Cases *n* (%)	Controls *n* (%)	OR (95%C.I)	*p* Value
Total	233	699		
Age (years)				<0.0001
≤40	11 (4.7%)	61 (8.7%)	1.00	
41–45	44 (18.9%)	187 (26.8%)	1.31 (0.63–2.68)	0.20
46–50	106 (45.5%)	282 (40.3%)	2.08 (1.06–4.11)	0.04
≥51	72 (30.9%)	169 (24.2%)	2.36 (1.18–4.75)	0.0057
Smoking				
No	193 (82.8%)	626 (89.6%)	1.00	
Yes	40 (17.2%)	73 (10.4%)	1.78 (1.17–2.70)	0.0070
Alcohol Consumption				
No	192 (82.4%)	624 (89.3%)	1.00	
Yes	41 (17.6%)	75 (10.7%)	1.78 (1.18–2.69)	0.0065
BMI (m/kg^2^)				
Normal	88 (37.8%)	422 (60.4%)	1.00	
Abnormal	145 (62.2%)	277 (39.6%)	2.51 (1.85–3.41)	<0.0001
TG (mg/dl)			1.00	
Normal	74 (31.8%)	528 (75.5%)		
High (>1000)	159 (68.2%)	171 (24.5%)	6.63 (4.79–9.18)	<0.0001
HDL (mg/dl)				
Normal	132 (56.7%)	540 (77.3%)	1.00	
Low (M: <40; F: <50)	101 (43.3%)	159 (22.7%)	2.60 (1.90–3.56)	<0.0001
LDL (mg/dl)				
Normal	194 (83.3%)	622 (89.0%)	1.00	
High (>150)	39 (16.7%)	77 (11.0%)	1.62 (1.07–2.47)	0.0228
Vegetable intakes				
71–100%	67 (28.8%)	279 (40.7%)	1.00	
51–70%	125 (53.6%)	263 (38.3%)	1.98 (1.41–2.78)	0.0001
<50%	41 (17.6%)	144 (21.0%)	1.19 (0.77–1.84)	0.3858
Creatinine (mg/dl)				
Normal (0.6–1.4)	221 (94.9%)	536 (96.9%)	1.00	
Abnormal (<0.6 or >1.4)	12 (5.2%)	17 (3.1%)	1.71 (0.80–3.65)	0.1629
Uric acid (mg/dl)				
Normal (2.5–7.5)	196 (84.1%)	434 (88.3%)	1.00	
Abnormal(<2.5 or >7.5)	37 (15.9%)	65 (11.7%)	1.42 (0.92–2.20)	0.12
Urine protein (g/L)				
Normal (<0.1)	208 (96.3%)	513 (97.5%)	1.00	
Abnormal (≥0.1)	8 (3.7%)	13 (2.5%)	1.52 (0.62–3.72)	0.3609
Diabetes mellitus				
No	221 (94.8%)	686 (98.1%)	1.00	
Yes	12 (5.2%)	13 (1.9%)	2.86 (1.29–6.37)	0.0099
Thyroid disease				
No	230 (98.7%)	693 (99.1%)	1.00	
Yes	3 (1.3%)	6 (0.9%)	1.51 (0.37–6.07)	0.5642

**Table 2:** Independent effect of *GNB3* gene on the risk of hypertension.

*GNB3*	Cases *n* (%)	Controls *n* (%)	OR (95%C.I)	aOR (95%C.I) *	*p* Value
Total	233	699			
CC + TC	160 (68.7%)	501 (71.7%)	1.00	1.00	
TT	73 (31.3%)	198 (28.3%)	1.15 (0.84–1.59)	1.21 (0.85–1.74)	0.2929

Note: * Logistic regression controlling for age, BMI, LDL, HDL, TG, diabetes mellitus, smoking and alcohol consumption.

**Table 3:** Independent effect of sodium intake on the risk of hypertension.

Sodium Intake	Cases *n* (%)	Controls *n* (%)	OR (95%C.I)	aOR (95%C.I) *	*p* Value
Total	233	699			
Healthy salt intake ≤ 2500 mg (roughly 1 teaspoon of salt)	182 (78.1%)	621 (88.8%)	1.00	1.00	
High salt intake > 5000 mg (roughly 2 teaspoons of salt)	51 (21.9%)	78 (11.2%)	2.23 (1.51–3.29)	1.88 (1.20–2.94)	<0.0001

Note: * Logistic regression controlling for age, BMI, LDL, HDL, TG, diabetes mellitus, smoking and alcohol consumption.

**Table 4:** Joint effect of *GNB3* gene and sodium intake on the risk of hypertension.

Combination of *GNB3* Gene and Sodium Intake	Cases *n* (%)	Controls *n* (%)	OR (95%C.I)	aOR (95%C.I) *	*p* Value
Total	233	699			
CC or TC + Healthy sodium intake	128 (54.9%)	446 (63.8%)	1.00	1.00	
CC or TC + High sodium intake	32 (13.7%)	55 (7.9%)	2.03 (1.26–3.27)	1.51 (0.88–2.60)	0.1916
TT + Healthy sodium intake	54 (23.2%)	175 (25.0%)	1.08 (0.75–1.55)	1.08 (0.72–1.61)	0.0114
TT + High sodium intake	19 (8.2%)	23 (3.3%)	2.88 (1.52–5.45)	3.23 (1.52–6.83)	0.0140
Attributable proportion due to interaction (AP)				0.51 (0.07–0.95)	
Rothman synergy index				3.79 (0.58–24.66)	

Note: * Logistic regression controlling for age, BMI, LDL, HDL, TG, diabetes mellitus, smoking, and alcohol consumption.

## Supplementary Materials

The following are available online at http://www.mdpi.com/1660-4601/15/6/1110/s1, Figure S1: Odds ratio with contributions from different *GNB3* gene polymorphisms or sodium intake categories marked focusing on non-smoking and non-alcoholic consumer; Table S1: Personal Characteristics of the Baseline Study Population that Lost-To Follow-up and the 10-year Cohort; Table S2: Joint Effect of *GNB3* Gene and Sodium Intake on the Risk of Hypertension focusing on non-smoking and non-alcohol consumption.

## Abbreviations

*GNB3*	protein beta3 subunit;
JNC7	Seventh Report of the Joint National Committee on Prevention, Detection, Evaluation, and Treatment of High Blood Pressure;
BP	blood pressure.
