# Supplementary material for: Interactions of Genes and Sodium Intake on the Development of Hypertension: A Cohort-Based Case-Control Study
Source: Int J Environ Res Public Health. 2018 May 30;15(6):1110. doi: 10.3390/ijerph15061110 (PMC6025596; doi:10.3390/ijerph15061110)
Supplement: Supplementary file 1 [file ijerph-15-01110-s001.docx]

Supplementary Material

Interactions of Genes and Sodium Intake on the Development of Hypertension: A Cohort-Based Case-Control Study

Mei-Ling Chen, Tzu-Pi Huang, Tai-Wei Chen, Hsin-Hua Chan and Bing-Fang Hwang

**Table S1.** Personal Characteristics of the Baseline Study Population that Lost-To Follow-up and the 10-year Cohort.

| Characteristic | Loss to follow up | 10-year cohort | *p*-value | Baseline |
| --- | --- | --- | --- | --- |
|  | N (%) | N (%) |  | N (%) |
| Number | 122 (100 %) | 1179 (100%) |  | 1301 (100%) |
| Age(year) |  |  |  |  |
| < 40 | 17(13.9%) | 124(10.5%) | 0.21 | 141(10.8%) |
| 41-45 | 39(32.0%) | 307(26.0%) |  | 346(26.6%) |
| 46-50 | 45(36.9%) | 466(39.5%) |  | 511(39.3%) |
| 51-55 | 13(10.7%) | 206(17.5%) |  | 219(16.8%) |
| > 55 | 8(6.5%) | 76(6.5%) |  | 84(6.5%) |
| Sex |  |  |  |  |
| Male | 102(83.6%) | 957(81.2%) | 0.51 | 1059(81.4%) |
| Female | 20(16.4%) | 222(18.8%) |  | 242(18.6%) |
| Smoking |  |  |  |  |
| No | 106(86.9%) | 1025(86.9%) | 0.99 | 1131(86.9%) |
| Yes | 16(13.1%) | 154(13.1%) |  | 170(13.1%) |
| Alcohol consumption |  |  |  |  |
| No | 107(87.7%) | 1032(87.5%) | 0.96 | 1139(87.6%) |
| Yes | 15(12.3%) | 147(12.5%) |  | 162(12.4%) |
| BMI (kg/m) |  |  |  |  |
| Normal | 47(38.5%) | 662(56.2%) | 0.01 | 709(54.5%) |
| Fat (>24) | 75(61.5%) | 517(43.8%) |  | 592(45.5%) |
| HDL (mg/dl) |  |  |  |  |
| Normal | 89(73.0%) | 853(72.4%) | 0.89 | 942(72.4%) |
| low  (M: <40; F: <50) | 33(27.0%) | 326(27.6%) |  | 359(27.6%) |
| LDL (mg/dl) |  |  |  |  |
| Normal | 102(83.6%) | 1039(88.1%) | 0.15 | 1141(87.7%) |
| high(>150) | 20(16.4%) | 140(11.9%) |  | 160(12.3%) |
| TG (mg/dl) |  |  |  |  |
| Normal | 82(67.2%) | 825(70.0%) | 0.53 | 907(69.7%) |
| high(>100) | 40(32.8%) | 354(30.0%) |  | 394(30.3%) |
| Vegetable intakes |  |  |  |  |
| 71-100% | 47(38.5%) | 411(35.7%) | 0.78 | 458(36.0%) |
| 51-70% | 47(38.5%) | 478(41.6%) |  | 525(41.3%) |
| <50% | 28(23.0%) | 261(22.7%) |  | 289(22.7%) |
| Creatinine (mg/dl) |  |  |  |  |
| Normal (0.6-1.4) | 84(96.6%) | 821(94.6%) | 0.43 | 905(94.8%) |
| Abnormal  (<0.6 or >1.4 ) | 3(3.4%) | 47(5.4%) |  | 50(5.2%) |
| Uric acid (mg/dl) |  |  |  |  |
| Normal(2.5-7.5) | 75(86.2%) | 763(87.8%) | 0.67 | 838(87.7%) |
| Abnormal  (<2.5 or >7.5) | 12(13.8%) | 106(12.2%) |  | 118(12.3%) |
| Urine protein(g/L) |  |  |  |  |
| Normal(<0.1) | 85(98.8%) | 800(97.4%) | 0.42 | 885(97.6%) |
| Abnormal(>0.1) | 1(1.2%) | 21(2.6%) |  | 22(2.4%) |

**Table S2.** Joint Effect of GNB3 Gene and Sodium Intake on the Risk of Hypertension focusing on non-smoking and non-alcohol consumption.

|  | **Cases**  **N (%)** | **Controls**  **N (%)** | **OR**  **(95%C.I)** | **aOR**  **(95%C.I)** | ***p* value** |
| --- | --- | --- | --- | --- | --- |
| Total | 173 | 573 |  |  |  |
| CC or TC + Healthy- sodium diets | 97  (56.1%) | 369  (64.4%) | R | R |  |
| CC or TC +High sodium diets | 19  (11.0%) | 46  (8.0%) | 1.57  (0.88-2.81) | 1.17  (0.61-2.23) | 0.33 |
| TT+  Healthy- sodium diets | 46  (26.6%) | 142  (24.8%) | 1.23  (0.83-1.84) | 1.30  (0.83-2.02) | 0.44 |
| TT+ High sodium diets | 11  (6.3%) | 16  (2.8%) | 2.62  (1.18-5.82) | 3.43  (1.38-8.58) | 0.02 |
| Attributable proportion due to interaction (AP) |  |  |  | 0.57  (0.12-1.03) |  |
| Rothman synergy index |  |  |  | 5.22  (0.46-57.20) |  |

Note: ^a^ Logistic regression controlling for age, BMI, LDL, HDL, TG and diabetes mellitus.

**Fig. S1.** Odds ratio with contributions from different GNB3 gene polymorphisms or sodium intake categories focusing on nonsmoking and nondrinking participants.
